# Impairing autophagy in retinal pigment epithelium leads to inflammasome activation and enhanced macrophage-mediated angiogenesis

**DOI:** 10.1038/srep20639

**Published:** 2016-02-05

**Authors:** Jian Liu, David A. Copland, Sofia Theodoropoulou, Hsi An Amy Chiu, Miriam Durazo Barba, Ka Wang Mak, Matthias Mack, Lindsay B. Nicholson, Andrew D. Dick

**Affiliations:** 1 School of Clinical Sciences, University of Bristol, Bristol, UK; 2 School of Cellular and Molecular Medicine, University of Bristol, Bristol, UK; 3 Department of Internal Medicine II, University Hospital Regensburg, Regensburg, Germany; 4 Institute of Ophthalmology, University College London, London, UK; 5 National Institute for Health Research (NIHR) Biomedical Research Centre, London, UK

## Abstract

Age-related decreases in autophagy contribute to the progression of age-related macular degeneration (AMD). We have now studied the interaction between autophagy impaired in retinal pigment epithelium (RPE) and the responses of macrophages. We find that dying RPE cells can activate the macrophage inflammasome and promote angiogenesis. *In vitro*, inhibiting rotenone-induced autophagy in RPE cells elicits caspase-3 mediated cell death. Co-culture of damaged RPE with macrophages leads to the secretion of IL-1β, IL-6 and nitrite oxide. Exogenous IL-6 protects the dysfunctional RPE but IL-1β causes enhanced cell death. Furthermore, IL-1β toxicity is more pronounced in dysfunctional RPE cells showing reduced IRAK3 gene expression. Co-culture of macrophages with damaged RPE also elicits elevated levels of pro-angiogenic proteins that promote *ex vivo* choroidal vessel sprouting. *In vivo*, impaired autophagy in the eye promotes photoreceptor and RPE degeneration and recruitment of inflammasome-activated macrophages. The degenerative tissue environment drives an enhanced pro-angiogenic response, demonstrated by increased size of laser-induced choroidal neovascularization (CNV) lesions. The contribution of macrophages was confirmed by depletion of CCR2^+^ monocytes, which attenuates CNV in the presence of RPE degeneration. Our results suggest that the interplay between perturbed RPE homeostasis and activated macrophages influences key features of AMD development.

Age-related macular degeneration (AMD) is a progressive ocular neurodegenerative disorder that leads to the loss of central vision. The disease is characterized by drusen and retinal pigment epithelial (RPE) abnormalities. In time the progression of AMD may result in either geographic atrophy (GA), where there is a contiguous area of RPE loss or neovascular AMD (nAMD)^1^. nAMD is characterized by choroidal neovascularization (CNV), a pathological angiogenesis arising from the vascular choriocapillaris resulting in the accumulation of fluid within the retina and subretinal space^1^, and if left untreated profound hemorrhage and scarring can cause irreversible visual loss. The complement system and immune related genes are widely accepted as a central driver to the progression of AMD^2^,^3^. However, the development of AMD is a slow process, and altered immune responses within the tissue likely occur as a result of persistent lifetime oxidative stress of RPE. Thus a combination of para-inflammation as well as heightened inflammasome activation and chronic inflammatory responses contribute to tissue destruction^4^,^5^,^6^.

Whilst GWAS can identify genetic polymorphisms associated with the risk of developing AMD, the main clinical hallmark and risk determinant remains observation of drusen accumulation at the interface between RPE and Bruch’s membrane (BM)^1^. Drusen are immunologically active deposits containing oxidative lipids, lipofuscin, complement and other immune activating components that develop as the consequence of RPE stress and altered tissue homeostasis^7^,^8^. Degenerating RPE is a major source for drusen components, indicating that age-related changes in RPE may be a causal factor and drive disease progression^9^.

Autophagy is the central cellular housekeeping function that facilitates the disposal of long-lived, defective organelles (eg. mitochondria) and protein aggregates through “self-eating” via autophagosomes and lysosomes^10^. Increasing evidence indicates impaired autophagy is associated with age-related degenerative disorders, highlighted by studies in which pharmacological or genetic manipulation of autophagy pathways can induce cellular and tissue degeneration *in vitro* and *in vivo*^11^,^12^,^13^. In the eye, autophagy is highly active in RPE and photoreceptor cells, and impaired autophagy in RPE leads to cell transcytosis and exocytosis and early signs of degeneration^13^,^14^,^15^. However, whether impaired autophagy pathways similarly influence immune responses within the tissue and contribute to the progression of AMD, is currently not known.

Although patients with AMD do not display signs of overt ocular inflammation, it is recognized that innate and adaptive immune responses contribute to the pathology of AMD^16^. The hallmarks of immune activation in ageing retina and choroid include macrophage accumulation and complement activation adjacent to and within drusen under the RPE, which is more pronounced in the presence of CNV^7^,^16^. One explanation for why patients convert from early AMD (drusen and mild RPE changes with autofluorescence) to late stage of AMD, is that triggers switch an ageing homeostatic para-inflammatory response to an unchecked persistent low grade inflammatory response resulting in loss of RPE and/or pathological angiogenesis^4^. We have recently demonstrated that RPE destruction in the model of laser-induced CNV polarizes infiltrating myeloid cells toward a pro-angiogenic phenotype. The latter can be perturbed through the augmentation of inhibitory CD200R signaling or through the administration of Th2 cytokines to either tonically suppress macrophage activation or drive anti-angiogenic function respectively^17^,^18^,^19^. Thus our data and those from others^20^,^21^ support the concept that interplay between macrophage and RPE within the subretinal space likely contributes to drusen formation and influences full disease progression. In the present study we demonstrate that impaired autophagy generates dysfunctional RPE that modulates macrophage responses, driving further cell death and promoting angiogenesis in the eye. These findings implicate that the interaction between degenerating RPE and subsequent macrophage activation simulates early events occurring in AMD leading to clinical expression and progression of neovascular disease.

## Results

### Inhibition of rotenone-induced autophagy results in caspase-3 mediated RPE cell death

As disease severity of AMD in patients progresses, there is an increase in damage of mitochondrial DNA (mtDNA), largely restricted to the RPE^22^. To establish an *in vitro* platform, we treated a murine RPE cell line (B6-RPE07)^23^ with rotenone (ROT), a mitochondrial complex 1 inhibitor to induce mtDNA damage^15^,^24^ (Supplementary Fig. 1A). ROT treatment (0.5 and 1 μM) resulted in altered mitochondrial function at 24 hours, as demonstrated by reduction in basal and stress induced oxygen consumption rate (OCR, indicator for mitochondrial respiration^25^). Treatment with the higher ROT concentration also led to decreased extracellular acidification rate (ECAR), a measure of glycolytic energy metabolism^25^, and therefore the lower 0.5 μM dose was selected for all further experiments. In response to mitochondrial damage, RPE cells showed enhanced autophagy and accumulation of LC3B^+^ autophagic vacuoles (Supplementary Fig. 1B). To inhibit autophagy, RPE cells were preincubated with wortmannin (WORT, 2 μM), an inhibitor that targets early events in autophagy cycle through irreversible inhibition of class III PI3-Kinase^12^. Although WORT is a potent PI3-Kinase inhibitor, its half-life in biological fluids is short ranging from 8 minutes to a maximum of two days depending on environmental factors^26^. However, WORT also exhibits prolonged anti-proliferative activity *in vitro* and induces neuronal degeneration after 2–7 days of treatment *in vivo*, suggesting prolonged biological consequences induced by initial WORT treatment^26^,^27^,^28^. After WORT pretreatment, the previously observed effects of ROT-induced autophagy were abolished (Supplementary Fig. 1B).

**Figure 1:**
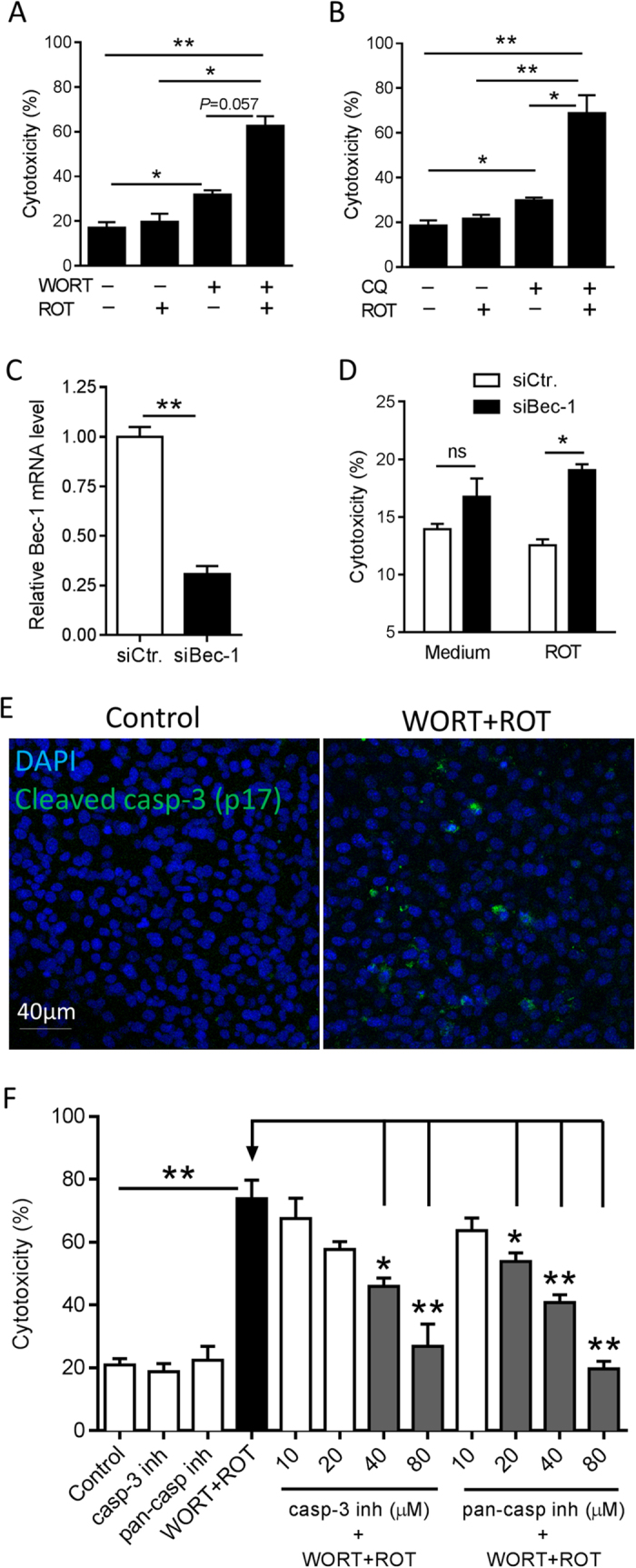
Inhibition of basal level or ROT-induced autophagy is toxic to RPE cell cultures. B6-RPE07 cells were pretreated with 2 μM WORT (**A**) or CQ (**B**) for 90 minutes, followed by further incubation with 0.5 μM ROT for 48 hours. Cytotoxicity was measured by LDH release in cell culture supernatants. (**C**) Real-time PCR analysis shows siRNA-mediated Beclin-1 knockdown in RPE cells. (**D**) Beclin-1 siRNA induces RPE cytotoxicity by ROT treatment post 48 hours. (**E**) Representative confocal images show expression of activated caspase-3 in RPE cells cultured with a combination of WORT and ROT for 24 hours, compared to the control. (**F**) LDH assay demonstrates dose-dependent effect of caspase-3 inhibitor or pan-caspase inhibitor on prevention of RPE death induced by WORT + ROT treatment. n ≥3. ^*^P < 0.05 and ^**^P < 0.01.

To assess the consequence of impaired autophagy on RPE with respect to cytotoxicity, we measured LDH release from dying/dead cells. ROT alone was not toxic to the cells after 48 hours (Fig. 1A), which was expected because increased autophagy serves as a protective mechanism for clearance of damaged mitochondria. Inhibition of the basal level of autophagy by WORT alone resulted in a mild increase in cytotoxicity, which was much less than the profound cytotoxicity (65%) that occurred as a result of autophagy inhibition by WORT followed by ROT-induced mitochondrial damage (normalized to LDH release in lysed RPE supernatant). These results were replicated in additional experiments (Fig. 1B) performed to confirm that RPE damage was mediated via impaired autophagy, but using chloroquine (CQ, 30 μM), another inhibitor of autophagy that prevents lysosomal acidification thus blocking down-stream autophagic degradation^12^. Using siRNA-mediated gene knockdown of Beclin 1, one of the key proteins involved in autophagosome formation^12^, led to increased LDH release from ROT-treated cells (Fig. 1C,D). Although Beclin 1 gene silencing elicited RPE susceptibility to ROT toxicity, the effect was less than that caused by the pharmacological inhibition (WORT or CQ) of autophagy, possibly because of continued operation of Beclin 1-independent autophagy pathways^29^.

Having established in this system that treatment with WORT impairs autophagy, it was then necessary to determine whether caspase-1 mediated pyroptosis^30^ or caspase-3 mediated apoptosis was evoked and contributed to RPE death. Immuno-staining revealed only caspase-3, but not caspase-1, activation in cells pretreated with WORT (Fig. 1E) or CQ (Supplementary Fig. 2) followed by ROT challenge, indicating apoptotic cell death. Experiments using either a caspase-3 specific inhibitor (Ac-DEVD-CHO), or pan-caspase inhibitor (Z-VAD-FMK) confirmed that this was a caspase-3 dependent effect, delivering a dose-dependent inhibition of RPE death (Fig. 1F). When used alone, neither of these inhibitors induced RPE death.

It is known that chronic photo-oxidative stress results in the production of reactive oxygen intermediates, leading to RPE and photoreceptor dysfunction and death^31^,^32^. As a comparator for RPE cytotoxicity caused by impairing autophagy we treated B6-RPE07 cells with the pro-oxidants hydrogen peroxide (H_2_O_2_) or paraquat (PQ). As expected, both compounds induced dose-dependent RPE death (Supplementary Fig. 3). Therefore, having established an *in vitro* platform in which degeneration of RPE can be induced by either dysfunctional autophagy or under oxidative stress, we then performed the following co-culture experiments to interrogate how macrophages respond to the RPE damage, in terms of inflammatory and angiogenic responses as seen in AMD.

### RPE dysfunction leads to macrophage inflammasome activation, inflammatory cytokine release and promotion of angiogenesis

**Figure 2:**
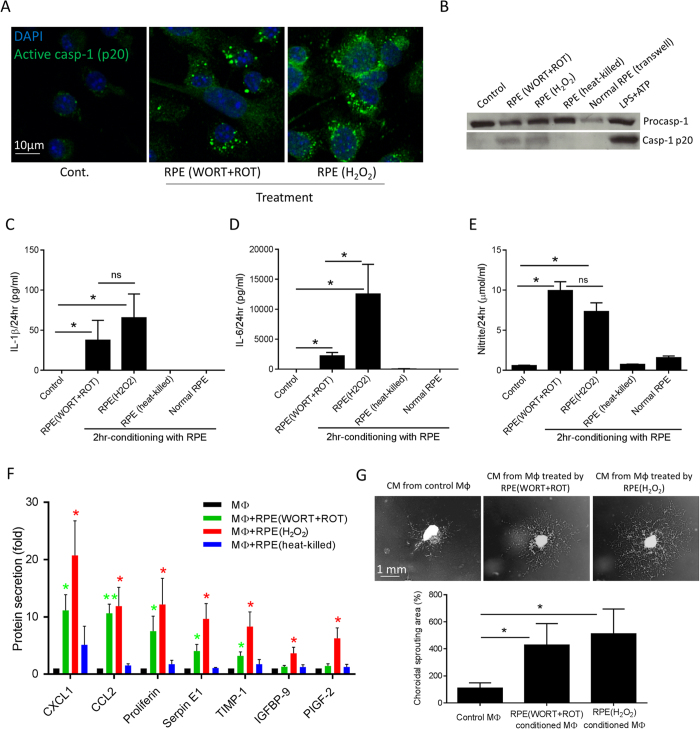
Autophagy-impaired and oxidative-stressed RPE cells induce BMMΦ inflammasome activation, inflammatory and angiogenic responses. BMMΦs were temporally incubated with differently damaged RPE cells/debris for 2 hours, before the un-engulfed RPE cells and debris were removed. Conditioned macrophages were further incubated in fresh cell culture medium for up to 24 hours. (**A**) Representative confocal images show the expression of activated caspase-1 p20 in BMMΦs conditioned by autophagy-impaired (WORT + ROT treated) or oxidative-stressed (H_2_O_2_ treated) RPE cells. (**B**) Cell culture supernatants from BMMΦs temporally conditioned by damaged RPE or co-cultured with normal RPE monolayers in transwell were analyzed for the secretion of cleaved caspase-1 by western blot. Treatment of LPS-primed BMMΦs with ATP for 60 minutes was used as a positive control for caspase-1 cleavage. RPE (heat-killed) refers to dead cell preparation heated at 95 °C for 15 minutes. ELISA was used to determine the production of IL-1β (**C**) and IL-6 (**D**) in BMMΦ culture supernatants. (**E**) Nitrite production from conditioned BMMΦs was detected using Griess reagent. (**F**) Mouse angiogenesis Proteome Arrays show the increased levels of selective angiogenesis-associated proteins secreted by conditioned macrophages. (**G**) Representative images show *ex vivo* choroidal neovascular sprouting after 6-day incubation with conditioned medium from BMMΦs. n ≥ 4. ^*^P < 0.05 and ^**^P < 0.01 vs. control or as indicated. ns, not significant.

To investigate how macrophages respond to the presence of dysfunctional RPE, bone marrow-derived macrophages (BMMΦs) were co-cultured with preparations of RPE damaged in different ways. In these assays, BMMΦs actively phagocytosed dying/dead RPE cells/debris within 60 minutes (Supplementary Fig. 4A). To assess the effects of BMMΦ conditioning by impaired RPE (either WORT + ROT treated or oxidative stressed with H_2_O_2_ treatment), damaged cells or cell fragments not engulfed by macrophages were removed by washing after 2 hours of co-culture (Supplementary Fig. 4B). The remaining adherent conditioned BMMΦs were then incubated with fresh cell culture medium for up to 24 hours. At this time, caspase-1 activation was induced (Fig. 2A) as was the secretion of capase-1 subunit p20, detected in the medium (Fig. 2B) of macrophages treated with either dysfunctional or stressed RPE, but not heat-killed (95 °C for 15 min) RPE or from macrophages co-cultured with normal RPE monolayers using transwell inserts. Treatment with dysfunctional or stressed RPE also led to the production of mature IL-1β which was detected in the supernatant of the conditioned macrophages (Fig. 2C), indicative of inflammasome activation. We noted that IL-18, a further product of inflammasome activation was not increased in macrophage supernatants, remaining at low levels (<8 pg/ml), which indicates possible dichotomous mechanisms of inflammasome processing of IL-1β and IL-18^33^. In addition to IL-1β, increased levels of IL-6 and nitric oxide (NO) production were also observed (Fig. 2D,E). Heat-killed RPE preparations or normal RPE monolayers (in transwell) did not induce cytokine and NO production from macrophages (Fig. 2C–E).

Given the pattern of inflammation and inflammasome activation, we next sought to determine whether the damaged RPE regulates the angiogenic potential of BMMΦs, and used a Proteome Profiler Array to examine 53 angiogenesis-associated proteins in the conditioned medium. The results showed varied upregulation of proteins from BMMΦs when treated with dysfunctional or stressed RPE as compared to control treated macrophages (Supplementary Fig. 5 and Fig. 2F). In total, there were seven macrophage-derived proteins that showed statistically significant changes over time when macrophages were co-cultured with damaged RPE cells for 2 hours (Fig. 2F). Macrophages conditioned with dysfunctional RPE cells produced increased levels of CXCL1, CCL2, proliferin, plasminogen activator inhibitor-1 (PAI-1 or Serpin E1) and tissue inhibitor of metalloproteinase 1 (TIMP-1), while macrophages conditioned with oxidative stressed RPE secreted higher amount of all of these proteins, as well as insulin-like growth factor binding protein 9 (IGFBP-9) and placental growth factor-2 (PlGF-2). Heat-killed RPE preparations did not significantly induce the production of any of these proteins from macrophages. There was no significant change in the level of VEGF detected in supernatants from macrophages following incubation with autophagy-impaired RPE cells. This array result was confirmed when the supernatants were applied to a VEGF ELISA (R&D Systems). We then examined the functional effect of activated BMMΦs on choroidal angiogenesis, using an *ex vivo* choroidal vascular sprouting assay^34^. Using this approach, choroidal explants seeded in Matrigel were incubated with medium from conditioned BMMΦs (Fig. 2G), and this demonstrated a pro-angiogenic effect of BMMΦ conditioned with either dysfunctional or stressed RPE.

### IL-1β and IL-6 differentially modulate RPE cell survival

**Figure 3:**
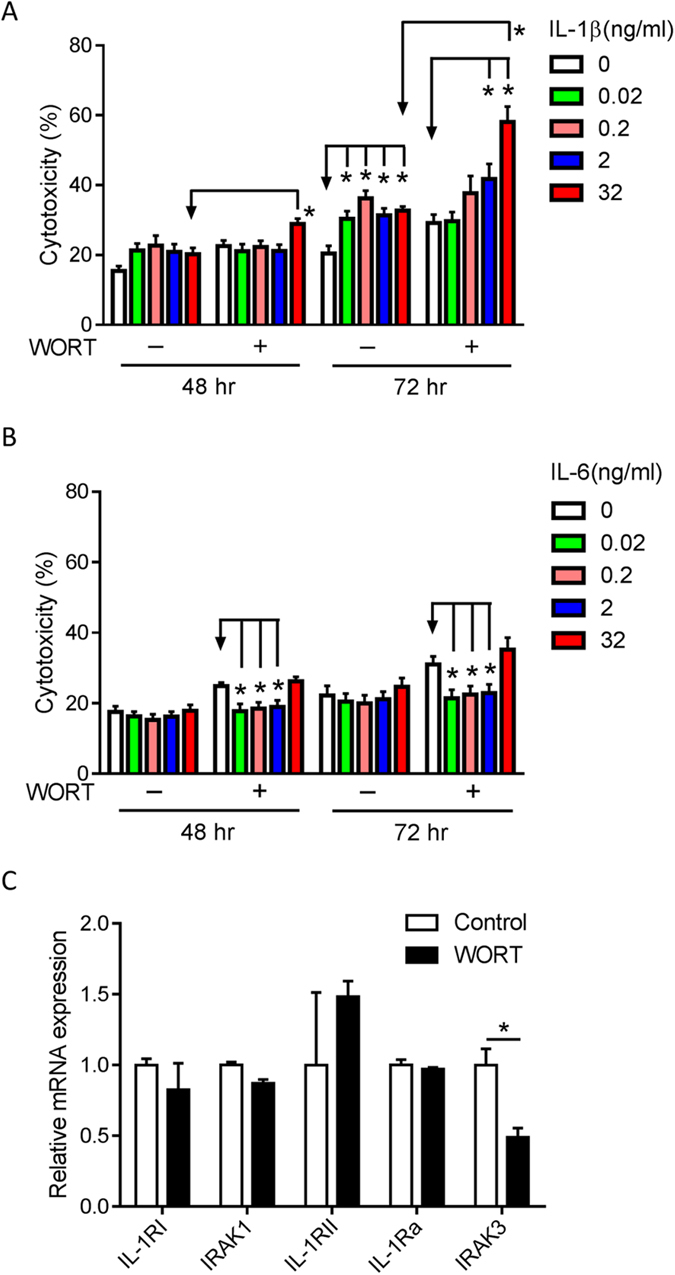
IL-1β and IL-6 variably influence RPE cell viability and the state of RPE is crucial for IL-1β cytotoxicity. RPE cells were treated with 2 μM WORT, different concentrations of IL-1β (**A**) or IL-6 (**B**), or a combination of WORT and the cytokine. Cytotoxicity was determined by LDH release at 48 and 72 hours, respectively. (**C**) RNA was isolated from RPE cells treated with WORT for real-time PCR analysis of gene expression associated with IL-1 signaling. n ≥ 3. ^*^P < 0.05.

Increased levels of inflammatory cytokines and chemokines have been observed systematically and in the eye of patients with AMD^35^,^36^,^37^. The data herein demonstrate that macrophages can produce such cytokines, including IL-1β and IL-6, in response to dysfunctional or degenerating RPE. The next question, therefore, is whether macrophage-derived cytokines maintain cellular homeostasis or induce death in normal or dysfunctional RPE. We assessed RPE cell cytotoxicity following treatment with WORT and/or increasing concentrations of the cytokines. After 48 hours of treatment (Fig. 3A), the highest concentration of IL-1β (32 ng/ml) induced cell damage in WORT-treated RPE cells compared with cells treated with IL-1β alone. However, by 72 hours even the lowest concentration of IL-1β (0.02 ng/ml) was toxic to RPE cells, and the higher doses (2 and 32 ng/ml) markedly amplified cytotoxicity in WORT-treated cells (Fig. 3A). In contrast, low concentrations of IL-6 (0.02–2 ng/ml) were protective against WORT-induced cytotoxicity after 48 and 72 hours (Fig. 3B). The data indicate a differential effect of IL-1β and IL-6 in damaging or protecting RPE respectively, and suggest that when autophagy is impaired RPE is more susceptible to IL-1β toxicity, compared with cells in which autophagy is maintained.

Supplementary to and corroborating the above data that demonstrate susceptibility of impaired RPE to cytokine, we further refined the findings in a mixed normal and dysfunctional RPE co-culture. Since the presence of WORT hampered the co-culture of damaged RPE with normal cells, the system exploited the knowledge that continuously cultured cells (for a prolonged period and post confluence) exhibit an altered cell cycle and susceptibility to death^38^ and which we could recapitulate in RPE. Unlike 3-day RPE cultures (Supplementary Fig. 6A,B), 6-day cultures demonstrated very strong nuclear staining of mitoSOX Red, indicative of loss of mitochondrial function^39^, and reduced autophagy (decreased LC3B^+^ fluorescent puncta in response to ROT treatment). A co-culture of 6-day cultured cells (labeled with CFDA) with 3-day cells (non-fluorescent) showed a preferential IL-1β treatment effect. Following IL-1β treatment, 50% of 6-day cells were absent from the monolayers (Supplementary Fig. 6C right panel and D). Under such conditions the remaining fluorescent 6-day cells were dead (Supplementary Fig. 6C right panel and E), indicating greater sensitivity to signals such as IL-1β inducing cell death compared to 3-day cells.

We next considered whether the increased susceptibility of dysfunctional RPE to IL-1β cytotoxicity may result from altered IL-1 signaling in these cells. To assess this, gene expressions of relevant molecules, such as IL-1 receptor type I (IL-1RI) and IL-1 receptor associated kinase 1 (IRAK1), which are required for IL-1 mediated NF-κB activation, and IL-1RII, IL-1R antagonist (IL-1Ra) and IRAK3, which downregulate IL-1 mediated inflammatory responses were assessed^40^,^41^. Impairment of autophagy in RPE cells with WORT resulted in a 50% reduction of IRAK3 gene expression compared with control cells (Fig. 3C), and in 6-day cultured cells expression of IRAK3 and IL-1Ra mRNA were both reduced by 90%, and other genes remained unchanged (Supplementary Fig. 6F). Combined this evidence suggests that in RPE where autophagy is impaired, expression of regulatory components of the IL-1 signaling pathways are altered, making cells more susceptible to IL-1β mediated cytotoxicity.

### Inducing impaired autophagy *in vivo* causes RPE and photoreceptor loss and subretinal accumulation of inflammasome-activated macrophages

**Figure 4:**
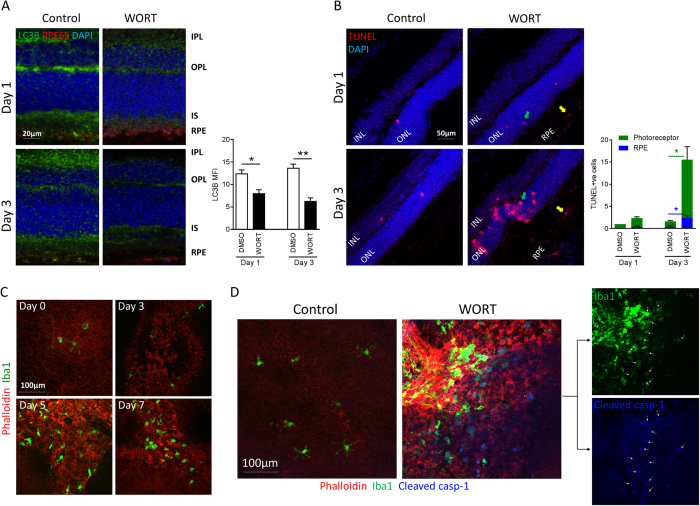
Inhibition of retinal autophagy causes RPE and photoreceptor death, followed by subretinal recruitment of inflammasome-activated macrophages. Mice were treated with WORT (0.5 mM in 2 μl PBS containing 2.5% DMSO) or control through intravitreal injection. Eyes were collected and sections or RPE/choroid whole-mounts were prepared at different time points. (**A**) Representative confocal images of sections show the expression of LC3B in the retina on days 1 and 3 post WORT injection (anti-RPE65 antibody was used to stain RPE). Quantitative MFI analysis demonstrates reduced LC3B immuno-positivity in retina. (**B**) Representative images show apoptotic cells (TUNEL stain) in the retina. Green arrows point to apoptotic cells in ONL and yellow arrows point to cells in RPE. TUNEL-positive photoreceptor and RPE cells per field were counted in three different sections from at least two mice at each time point. (**C**) Representative images of RPE/choroidal whole-mounts show alteration of F-actin organization on RPE monolayer (phalloidin stain) and recruitment of macrophages (Iba1 stain) at different days post WORT injection. (**D**) Representative images show the expression of cleaved caspase-1 predominantly on recruited Iba1^+^ cells at site of RPE degeneration, but not from RPE. White arrows point to cells expressing both Iba1 and cleaved caspase-1 p20. Ramified microglia distributed on the normal RPE do not express activated caspase 1. n ≥ 4. ^*^P < 0.05, ^**^P < 0.01.

*In vitro* assessment demonstrates that impaired autophagy in RPE cell culture causes RPE apoptosis that in turn modulates macrophage-inflammasome activation. To confirm this *in vivo*, we impaired autophagy via intravitreal injection of WORT (0.5 mM in 2 μl PBS containing 2.5% DMSO), and with an equal volume of PBS containing 2.5% DMSO as the control in the contralateral eye. As shown in Fig. 4A, naïve or control sections displayed strong expression of LC3B within the inner plexiform layer (IPL), outer plexiform layer (OPL), inner segment (IS) and RPE (RPE-65 stain), representing retinal cells with high metabolic demand^14^. Following WORT administration, there was a rapid decrease in autophagy activity supported by reduced LC3B expression after 1 day, and this was more pronounced by day 3 (Fig. 4A). Similar to neuronal function^42^, WORT attenuation of retinal autophagy was transient as we noted the LC3B expression reversed after 7 days (Supplementary Fig. 7). Contemporaneous to the early reduction in autophagy activity, there was an increase in TUNEL-positive apoptosis detected within ONL and RPE by day 3 (Fig. 4B). Our observation that photoreceptor and RPE cells are more susceptible to WORT-induced cell death compared to other retinal cells supports a critical role of autophagy in sustaining photoreceptor and RPE health and maintaining visual function.

We next examined how WORT administration and impaired autophagy influence pathologic changes to the RPE and subretinal space. Following RPE morphology on RPE/choroidal flat-mounts through time, disorganised F-actin expression and loss of honeycomb-like structure in RPE monolayer can be observed by day 3 post-WORT administration (Fig. 4C), and the subsequent accumulation of Iba1^+^ macrophages by day 5 (Fig. 4C and Supplementary Fig. 8). Furthermore, cleaved caspase-1 p20 immuno-staining demonstrated that caspase-1 activation was restricted to the subretinal macrophages, and was absent in the ramified microglia sparsely distributed on the retinal surface of normal RPE (Fig. 4D). Notably, the strongest signal for cleaved caspase-1 was restricted to subretinal macrophages, instead of the RPE (Fig. 4D and Supplementary Fig. 9).

### CCR2^+^ monocyte-derived macrophage dependent laser-induced CNV is augmented in the presence of dysfunctional RPE

**Figure 5:**
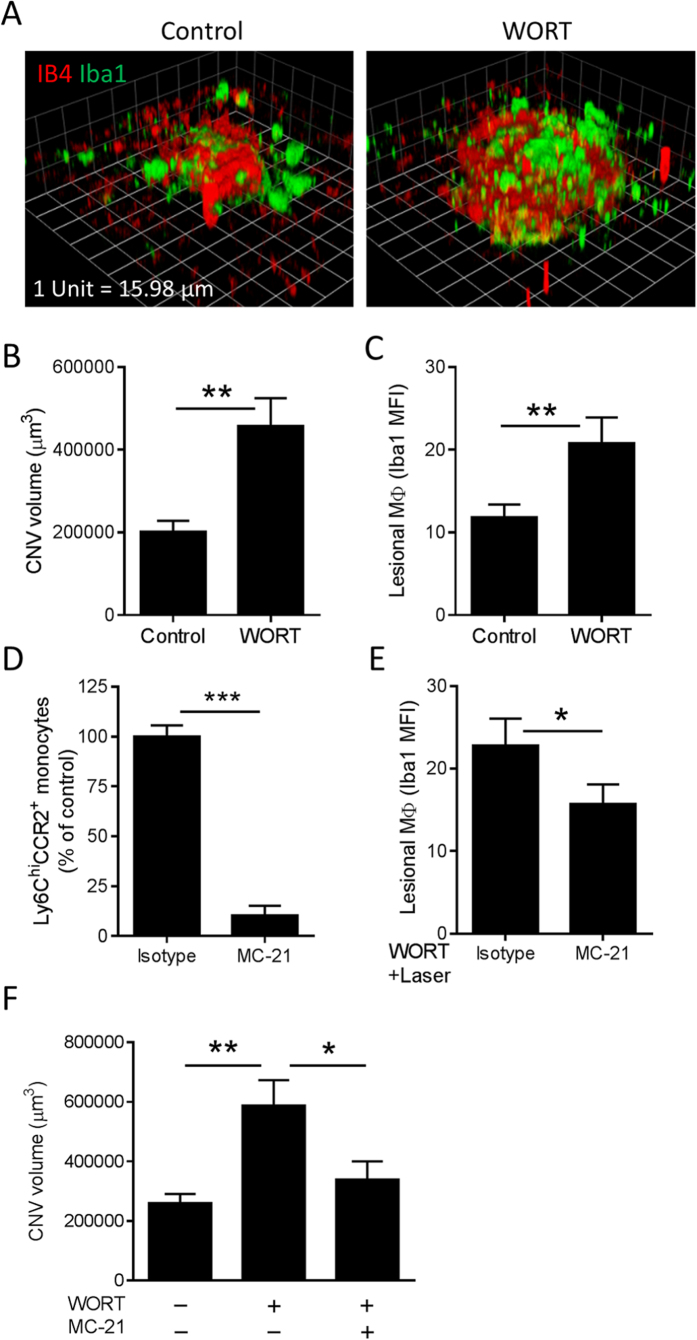
Contribution of RPE degeneration and infiltrating CCR2^+^ monocytes to laser-induced CNV in mice. Five days after intravitreal injection of WORT, laser photocoagulation was induced in mice. On day 7 post laser induction, RPE/choroidal whole-mounts were processed and stained with IB4 and anti-Iba1 for assessment of neovascular membrane and macrophage accumulation, respectively. (**A**) Representative images show 3D reconstructions of CNV lesions captured by confocal microscopy and analyzed by Volocity Imaging software. Quantitative analysis shows that the WORT-induced RPE pathology exaggerates CNV volume (**B**) and lesional macrophages (**C**) compared to control. (**D**) Flow cytometric analysis confirms significant depletion of peripheral Ly6C^hi^ CCR2^+^ monocytes by ip. injection of anti-CCR2 mAb (MC-21), compared with the rat IgG2b isotype. (**E**) MFI analysis using ImageJ software demonstrates reduced macrophage accumulation at CNV lesions by MC-21 injection. (**F**) Comparison of CNV volume shows abolishment of WORT induced laser-CNV by depletion of CCR2^+^ monocytes. (**B,C,E,F**) n ≥ 24. (**D**) n = 8. ^*^P < 0.05, ^**^P < 0.01, ^***^P < 0.001.

By day 12 post WORT injection the disorganised morphology of RPE was reversed and equivalent to control animals with no evidence of spontaneous CNV (Supplementary Fig. 10), precluding longer term experimentation in animals. Nevertheless, our *in vitro* findings demonstrating macrophage pro-angiogenic function induced by dysfunctional RPE and the *in vivo* evidence of transient impaired autophagy and RPE dysregulation prompted us to study whether under such conditions we could augment pathological angiogenesis. We therefore combined the WORT-induced RPE degeneration with a laser-induced CNV model. At day 5 post-WORT administration, where macrophage recruitment is maximal, laser photocoagulation was applied to disrupt BM and induce CNV. Seven days post laser induction (ie. 12 days after drug injection), isolectin B4 (IB4) and Iba1 co-staining demonstrated that the CNV volume was significantly increased in animals treated when autophagy was impaired and macrophage infiltration was present (Fig. 5A,B). There was also an increase in Iba1 immuno-reactivity within and adjacent to CNV lesions (Fig. 5A,C).

Given the assumption that by day 5 the macrophage accumulation was likely infiltrating cells we wished to ask whether as we have previously shown^17^, the inflammatory CCR2^+^ monocytes play a role in accentuating laser-induced CNV on the background of RPE dysfunction through impaired autophagy. To answer this, the CCR2^+^ monocyte subset was depleted using a daily MC-21 mAb^17^,^43^ treatment regime (days 4–8 post-WORT treatment). Depletion efficiency was assessed by FACS analysis of peripheral blood samples and confirmed a significant reduction of Ly6C^hi^ CCR2^+^ monocytes (Fig. 5D). MC-21 treatment also resulted in a reduction of Iba1^+^ lesional macrophages when compared with isotype controls (Fig. 5E), and depletion of the inflammatory CCR2^+^ monocyte subset reversed the RPE dysfunction mediated accentuation of laser-CNV (Fig. 5F).

## Discussion

Clinical and experimental evidence supports that in part the pathogenesis of AMD involves the interplay of RPE dysfunction, macrophage recruitment and immune activation. There remains a need to understand early pathological events and the mechanisms at play, such as what induces a switch driving cytopathic or angiogenic macrophage responses and enhancing progression of pathology. Here our data suggest a mechanism for such a switch: RPE cells, rendered dysfunctional through impairing autophagy, are capable of inducing inflammasome activation in macrophages, amplifying inflammatory responses and promoting angiogenesis.

As a homeostatic mechanism, autophagy is active in young retina/RPE and such activity increases with age, in part, due to age-related accumulation of damaged organelles and intracellular waste^14^,^15^. Defective autophagy is also associated with age-related degenerative conditions, and for example in RPE via failure in clearance of ELAV1/HuR-mediated aggregation of SQSTM1/p62 protein that is normally degraded by proteasomes in healthy RPE^44^. Particularly, mtDNA damage is predominant in RPE from patients with AMD^22^, and enhances oxidative damage to the cells^45^. We demonstrate that impairing RPE autophagy and clearance of damaged mitochondria leads to caspase-3 dependent apoptosis. Inhibition of caspase-3 can promote cell survival despite impaired autophagy. Notwithstanding, RPE cells also undergo caspase-1 mediated pyroptosis by lysosomal destabilization^30^. Therefore the cell death pathways implicated in RPE degeneration are conditional and dependent on the exact pathological drive.

The inflammasome is a canonical innate immune response, from response to signals through pathogen-associated molecular patterns (PAMPs) during infection or endogenous cellular and tissue damage-associated molecular patterns (DAMPs)^46^. Recent advances also reveal that NLRP3 inflammasome can act like a metabolic sensor in response to metabolic alterations in cells, and its activation is associated with several metabolic disorders, such as type 2 diabetes and obesity^47^. Accumulating evidence unequivocally demonstrates an association between inflammasome activation and development or regulation of many of the age-related degenerative disorders, including atrophic and nAMD^5^,^48^,^49^. In particular, activation of NLRP3-inflammasome in RPE cells has been purported as a causal factor for RPE dysfunction and cell death^30^,^50^. We take the observations further, showing that inflammasome activation occurs in infiltrating macrophages in the subretinal space and in response to RPE degeneration evoked by impaired autophagy. Tissue flat-mounts (Fig. 4D) show that the strongest expression of cleaved caspase-1 p20, a marker for inflammasome activation, is found in recruited macrophages, rather than damaged or surrounding RPE.

In this study, our *in vitro* evaluation shows that IL-1β and IL-6 differentially influence RPE cell viability in a dose- and time-dependent manner, and is dependent on pretreated health of the RPE. What was revealed is that IL-6 (≤2 ng/ml) protects against damage to dysfunctional (impaired autophagy) RPE cells, whilst IL-1β is largely cytotoxic with time although we do not know if this is secondary to cytokine accumulation or not. It was recently identified that an activation of MyD88-mediated IRAK1/4 induced by RNase DICER1 deficiency leads to GA-like RPE degeneration^49^, although whether there is a change in negative regulator(s) for IL-1β signaling has not been previously studied in the context of AMD. Our present data show a reduced gene expression of IRAK3 and/or IL-1Ra in abnormal RPE including impairment in autophagy and continuously cultured cells. Indeed, such RPE cells are more susceptible to IL-1β induced cytotoxicity than normal cell cultures. Future studies are warranted to test whether recovery of the regulatory mechanism could have beneficial effects for RPE survival. It will be important to further interrogate how targeting IL-1β or IL-6 may influence RPE degeneration and choroidal angiogenesis in the presence of impaired autophagy *in vivo*.

We also found that the lowest concentration (ie. 20 pg/ml) of either IL-1β or IL-6 still influenced the RPE viability, consistent with the observation that very low levels of circulating cytokines (generally <100 pg/ml despite variation among patients) can affect brain function^51^. Although the high cytokine doses we used (32 ng/ml) are unlikely to exist in the circulation or within ocular fluids of AMD patients^35^, we cannot exclude local high concentration of cytokines at the site of damage as shown in model of traumatic injury^52^. Although cytokines demonstrate pleiotropism, studies have shown that IL-6 protects ganglion cells against pressure-induced apoptosis^53^, and promotes recovery of murine CNS following brain injury^54^. This has to be squared with the findings that serum concentrations of IL-6 correlate positively with higher risk for AMD^55^ and IL-6 autocrine signaling induces RPE degeneration following lipopolysaccharide stimulation^56^.

Macrophages are pivotal to angiogenesis during development, tissue injury and regeneration. Studies have demonstrated that macrophages can both promote and suppress angiogenesis irrespective of phenotype signatures, but cells adapt according to environmental stimuli^17^,^18^,^19^,^57^. Previous studies have focused on oxidative stress-induced retinal degeneration or angiogenesis caused by reagents that generates oxygen radicals, or under long-term exposure of photic stress^31^,^32^. Refining the models to specifically answer questions as to whether mitochondrial stress and impaired autophagy drive specific macrophage responses, we can now provide evidence indicating that RPE cells with impaired autophagy promote angiogenesis in presence of disrupted Bruch’s membrane, but dependent on CCR2^+^ macrophages providing requisite angiogenesis-associated proteins. These proteins from the conditioned macrophages include chemokines (CXCL1^58^ and CCL2^59^), proteins that regulate endothelial cell migration (proliferin^60^) and matrix remodeling (Serpin E1^61^ and TIMP-1^62^), all of which could directly drive neovascularization.

IGF-1, a downstream effector of growth hormone, has been observed in human CNV lesions and patients with nAMD have increased aqueous humor IGF-1 and IGFBP-2^63^. RPE-VEGF expression can be induced by IGF-1 *in vitro*^64^. As a member of the VEGF family, PlGF is also found in human CNV and promotes laser-induced CNV in mice. Here we did not find upregulation of any proteins related to IGF or PlGF signaling in macrophages treated with autophagy-impaired RPE, but macrophages conditioned by oxidative stressed RPE exert increased IGFBP-9 and PlGF-2. Our observation that dysfunctional autophagy and oxidative stress induced RPE death have both distinct and overlapping effects that modulate macrophage phenotypes involved in inflammation and angiogenesis (Fig. 2), is consistent with the findings that these two mechanisms cross talk via redox reaction and metabolic network^65^.

Retinal capillary and neuron degeneration by intraocular treatment with WORT has been reported to be an acute pathologic condition in animals occurring within 2–7 days of treatment, and long-term effects have not been investigated^27^,^28^. Here we show that WORT-induced RPE degeneration and the ensuing macrophage activation do occur within a similar timeline of 2–7 days but subside by day 12. So we were unable to generate a longer-term model of insidious pathologic changes to simulate AMD processes. Nevertheless we were able to harness the model to demonstrate early RPE response to impaired autophagy and then exploit the model to supplement the effect on laser-induced CNV. Laser-induced CNV induces BM breakdown allowing for a ‘wound-healing’ neovascularization responses. We note that in the presence of RPE dysfunction, CNV is exaggerated and dependent upon inflammatory infiltrating macrophages. This is supported by observations in *Ccl*2- or *Ccr*2-knockout mice^66^, our previous data refining the role of macrophages in laser-induced CNV^17^,^18^ and elevated intraocular CCL2 level in patients with nAMD^36^. Our current data further our understanding: Firstly, there is a recruitment of monocyte/macrophages when RPE are rendered dysfunctional through impaired autophagy, which are activated to express pro-inflammatory and pro-angiogenic proteins and secondly, CNV formation is augmented and dependent upon inflammatory macrophages.

In summary, our results show that impairing autophagy induced RPE degeneration modulates infiltrating macrophage activation, resulting in inflammation induced cytotoxicity and angiogenesis. *In vitro* data show that the inflammatory cytokines IL-1β and IL-6 differentially influence RPE cell survival, where dysfunctional RPE cells are more susceptible to IL-1β cytotoxicity. We suggest that the overall state of RPE health and the extent of inflammatory responses contribute to the development of AMD at early clinical stages.

## Methods

### Cell culture and treatment

A spontaneously transformed mouse RPE cell line B6-RPE07^23^ was kept in DMEM medium supplemented with 10% heat-inactivated fetal calf serum, 2% L-glutamine, 1 mM sodium pyruvate, 60 μM 2-mercaptoethanol, 100 U/ml penicillin and 100 μg/ml streptomycin (complete medium) at 37 °C in an atmosphere of 5% CO_2_. RPE cells were passaged with a split ratio of 1:5 using 0.05% Trypsin-EDTA (Life Technologies, Paisley, UK), and allowed to recover for 2 days in complete medium prior to experiments. Confluent B6-RPE07 cell cultures exhibit cobblestone-like morphology similar to primary mouse RPE cultures. The cells also express RPE markers, tight junction and adhesion molecules, and show polarized phenotype when cultured in collagen-coated membrane^23^ and have been used as an *in vitro* RPE model in many recent functional studies^17^,^18^,^67^.

For induction of dysfunctional RPE, cells were seeded at a density of 1 × 10^5^/cm^2^ in cell culture plates to form a confluent monolayer during an overnight incubation. All chemical inhibitors or damage inducers were purchased from Sigma-Aldrich, Poole, UK unless otherwise stated. RPE autophagy was inhibited by the addition of wortmanin (WORT, 2 μM) or chloroquine (CQ, 30 μM) (Life Technologies) for up to 48 hours. Rotenone (ROT, 0.5–1 μM) was used to induce mtDNA damage through inhibition of mitochondria complex 1 function. In some experiments, RPE cells were pretreated with either a caspase-3 inhibitor (Ac-DEVD-CHO, 10–80 μM) or pan-caspase inhibitor (Z-VAD-FMK, both from Enzo Life Sciences, Exeter, UK) before addition of WORT and ROT. Oxidative stress was induced by the addition of H_2_O_2_ (1–2 mM) or paraquat (PQ, 0.25–2 mM). Cultures of normal or dysfunctional (WORT-treated) RPE cells were also stimulated by various concentrations of recombinant cytokines IL-1β or IL-6 (0.02–32 ng/ml, PeproTech, London, UK).

Bone marrow derived macrophages (BMMΦs) were generated using a previously described method^17^. In brief, bone marrow cells were washed in DMEM media and resuspended at 1 × 10^5^ cells/ml in complete medium supplemented with 5% horse serum (GE Healthcare Life Sciences, Little Chalfont, UK) and 100 pg/ml macrophage-colony stimulating factor (M-CSF). Cell suspensions (50 ml) were transferred to Teflon-coated tissue culture bags (produced in-house) and incubated for 8 days at 37 °C in 5% CO_2_. Cells were removed from the Teflon bags and plated at 1 × 10^6^ cells/well in 24-well plates (Corning, Flintshire, UK), and incubated for 2 hours to allow cell attachment. During this 2-hour period, damaged RPE cells were collected and washed three times in serum-free DMEM medium to remove free drug. Cell culture supernatant of adherent macrophages was then replaced by serum-free medium containing 1 × 10^6^ damaged RPE cells. Damaged RPE cells and debris lose their ability to attach to cell culture plates within 2 hours of incubation and are readily removed following washes with cell culture media. After 2 hours of co-culture cells were washed to remove un-engulfed RPE cells/debris, before addition of fresh cell culture media to macrophages for final incubation of 24 hours. Cell supernatants were collected for protein analysis using ELISA, Angiogenesis Proteome Array or Western blot.

### Antibodies

Mouse anti-caspase-1 mAb (AG-20B-0042), which recognizes both full-length and cleaved p20 fragments, was purchased from Caltag Medsystems (Buckingham, UK). Goat polyclonal anti-cleaved caspase-1 p20 was obtained from Santa Cruz Biotechnology (Heidelberg, Germany). Rabbit anti-cleaved caspase-3 mAb (5A1E), HRP conjugated goat anti-rabbit and anti-mouse IgG were from New England Biolabs (Hitchin, UK). Rabbit polyclonal anti-LC3B, mouse anti-RPE65 mAb (401.8B11.3D9), DyLight^®^ 488 conjugated donkey anti-rabbit IgG and Alexa Fluor 405-donkey anti-goat IgG were from Abcam (Cambridge, UK). Rabbit polyclonal anti-Iba1 was obtained from Wako Chemicals (Neuss, Germany). Alexa Fluor 488 conjugated rabbit anti-goat IgG, Alexa Fluor 488 conjugated goat anti-rabbit IgG and Alexa Fluor 555 conjugated goat anti-mouse IgG were from Life Technologies.

### *In vitro* assessment of RPE damage

At various time-points RPE cell culture supernatants were collected and chemical-induced RPE cytotoxicity was assessed using a LDH detection kit (Abcam, Cambridge, UK) according to manufacturer’s instructions. Activity of released LDH was normalized to the value of RPE lysates (100% cytotoxicity).

Caspase-3 activation in RPE cells was detected by immuno-staining. Briefly, drug-treated cells were fixed with 2% (wt/vol) paraformaldehyde (PFA) and permeabilized with 0.1% Triton X-100. After blocking with 5% BSA, the cells were incubated with a rabbit mAb against cleaved caspase-3 (Asp175) (1:400, clone 5A1E, New England Biolabs, Hitchin, UK) overnight at 4 °C, followed by incubation with a DyLight^®^ 488 conjugated donkey anti-rabbit IgG (1:200). The cells were then mounted with Vectashield antifade medium (Vector Laboratories, Peterborough, UK) and examined using a Leica TCS-SP2-AOBS confocal laser scanning microscope.

The expression of autophagic vacuoles was detected using a PremoTM Autophagy Sensor GFP-LC3B BacMam Kit (Life Technologies) according to manufacturer’s instructions. Briefly, B6-RPE07 cells were transduced with BacMam GFP-LC3B at a MOI of 30 in complete medium overnight, before the addition of chemicals for 24 hours. Cell monolayers were analyzed for the presence of LC3B-positive puncta using fluorescence microscopy.

RPE cell metabolic function was assessed by a Seahorse XFp Extracellular Flux Analyzer (Seahorse Bioscience Europe, Copenhagen, Denmark). Confluent B6-RPE07 cells were treated with ROT for 24 hours or left untreated. The cells were collected and seeded on XFp miniplates (5 × 10^5^ cells/well) by centrifugation at 800 × g for 10 minutes to allow quick adherence. Basal and stress-induced levels of oxygen consumption rate (OCR) and extracellular acidification rate (ECAR) were measured before and after injection of cell stress reagents, including carbonyl cyanide 4-(trifluoromethoxy)phenylhydrazone (FCCP, mitochondrial respiration inhibitor) and oligomycin (ATP synthase inhibitor), respectively.

To detect mitochondrial superoxide in live cells, MitoSOX Red (Thermo Fisher Scientific, Paisley, UK) was added at a final concentration of 5 μM according to manufacturer’s instructions. Cells were allowed to load MitoSOX for 10 min and washed twice with Hank’s Buffered Salt Solution (HBSS) containing calcium and magnesium. Hoechst 33342 (Life Technologies) was used for counterstain of nuclei. Cells were then observed using confocal microscopy.

### Beclin 1 RNAi

To knockdown Beclin 1 expression in B6-RPE07 cells, the FlexiTube GeneSolution (GS56208, Qiagen, Manchester, UK), as a specific mixture of four preselected siRNA duplexes to target different sequences of the mouse Beclin 1 gene was utilized according to the manufacturer’s instructions. The siRNAs were mixed with HiPerFect siRNA transfection reagent (Qiagen) to form the transfection complex, prior to addition to RPE culture medium at a final concentration of 40 nM. Non-silencing siRNA (SI03650325, Qiagen) with the same concentration was used as a negative control. Beclin 1 expression was determined by western blot and real-time RT-PCR.

### Western blot

To detect BMMΦ secretion of caspase-1 proteins, serum-free conditioned media from BMMΦs were collected and separated by SDS-PAGE gel electrophoresis, and proteins transferred to a nitrocellulose membrane. Following blocking in 5% milk/TBS/Tween-20, the membrane was subjected to Western blot analysis using a mouse anti-caspase-1 mAb (1:1000). Proteins were detected with a HRP conjugated polyclonal anti-mouse IgG (1:2000) and visualized using the chemiluminescent method (GE Healthcare Life Sciences).

### Real-time RT-PCR

Total RNA from RPE cells was isolated using TRIzol reagent (Life Technologies), treated with RQ1 RNase-free DNase before cDNA synthesis using the ImProm-II^TM^ Reverse Transcription System (Promega, Southampton, UK). cDNA was amplified using the Power SYBR® Green PCR Master Mix Reagent (Life Technologies) on a StepOne™ Applied Biosystems Real-Time PCR System. Primer sequences used were: β-actin, forward 5′-AGC CAT GTA CGT AGC CAT CC, reverse 5′-CTC TCA GCT GTG GTG GTG AA; Beclin 1, forward 5′-CAG GAA CTC ACA GCT CCA TTA C, reverse 5′-CCA TCC TGG CGA GTT TCA ATA; IL-1RI, forward 5′-AGG TGG AGG ACT CAG GAT ATT, reverse, 5′-CCA GGG TCA TTC TCT AAC ACA G; IL-1RII, forward 5′-CTG ATA GTC CCG TGC AAA GT, reverse 5′-GGG TAA GCA GCC GAG ATA AA; IL-1Ra, forward 5′-TTG TGC CAA GTC TGG AGA TG, reverse 5′-CTC AGA GCG GAT GAA GGT AAA G; IRAK1, forward 5′-CAG AGG TGG AAC AGC TAT CAA G, reverse 5′-CAT TGG GCA AGA AGC CAT AAA C; and IRAK3, forward 5′-CAA CAA AGC CCA CCA TCA TTA C, reverse 5′-GCA TTT GAG CAA CTT CCC TAT G.

### *In vitro* assessment of macrophage responses

To assess caspase-1 activation in BMMΦ, cells were fixed and permeabilized before immuno-staining with a polyclonal goat anti-cleaved caspase-1 p20 antibody (1:50) followed by secondary detection using an Alexa Fluor 488 conjugated rabbit anti-goat IgG (1:400). Samples were then mounted and examined by confocal microscopy. In other experiments, serum-free conditioned media from BMMΦs were used for Western blot analysis of caspase-1 cleavage.

IL-1β and IL-6 concentrations in the conditioned BMMΦ supernatants were measured by sandwich ELISA kits (R&D Systems, Abingdon, UK). IL-18 concentration was detected by a Platinum ELISA kit (eBioScience, Hatfield, UK). Nitric oxide production in conditioned supernatant of BMMΦs was determined by the Griess reagent (Sigma-Aldrich) according to manufacturer’s instructions. .

To determine expression profiling of proteins associated with angiogenesis, BMMΦ supernatants were collected and probed with a mouse Angiogenesis Proteome Profiler Array kit (R&D systems) according to manufacturer’s instructions.

### *Ex vivo* choroid sprouting assay

Pro-angiogenic effect of conditioned medium from BMMΦs was assessed using a choroid sprouting assay^34^. Mouse peripheral choroid-scleral complex was dissected from C57BL/6J mice aged 6 to 8-week-old and cut into approximately 1 × 1mm pieces before being placed in Matrigel (BD Biosciences, Oxford, UK). Matrigel was used to coat the bottom of 48-well-plate without touching the edge of the well. After seeding the choroid segments, the plate was incubated at 37 °C for 10 minutes to solidify the Matrigel. 200 μl of conditioned medium was then added to each well and incubated at 37 °C with 5% CO_2_. Conditioned medium was replaced every 2 days. Phase contrast photos of each explant were taken on day 7 using Widefield imaging system microscope (Leica), and the sprouting areas were quantified using ImageJ 1.46r (National Institutes of Health, USA).

### Mice and *in vivo* experimental procedures

C57BL/6J mice were obtained from the Jackson Laboratory and breeding colonies were established within the Animal Services Unit at University of Bristol, UK. Mice were kept in the animal house facilities of the University of Bristol, according to the Home Office Regulations. Treatment of the animals conformed to the Association for Research in Vision and Ophthalmology (ARVO) statement for the Use of Animals in Ophthalmic and Vision Research. The methods were carried out in accordance with the approved University of Bristol institutional guidelines and all experimental protocols under a Home Office Project Licence 30/2745 were approved by the University of Bristol Ethical Review Group.

Mice aged 6–8 weeks were anesthetized by intraperitoneal (i.p.) injection of 200 μl of Vetelar (ketamine hydrochloride 100 mg/ml, Pfizer, Sandwich, UK) and Rompun (xylazine hydrochloride 20 mg/ml, Bayer, Newbury, UK) mixed with sterile water in the ratio 0·6:1:84. Pupils were dilated using topical 1% tropicamide. Local administration of WORT (0.5 mM in 2 μl PBS containing 2.5% DMSO) or vehicle control was performed by intravitreal injection using a 33-gauge hypodermic needle.

On day 5 post intravitreal injection of WORT, CNV was induced by laser photocoagulation in mice as previously described^17^. Four laser spots were delivered to the posterior retina using an OculightSlx Krypton Red Laser system (power 200 mW, duration 75 ms, spot size 75 μm). In selected experiments designed to assess the contribution of CCR2^+^ monocytes to CNV on a degenerative (WORT-induced) RPE background, mice were treated with an anti-CCR2 depleting mAb, MC-21^43^. A treatment regime commencing 24 hours before CNV induction, involved daily i.p. injections of MC-21 (20 μg/dose) or an isotype control (rat IgG2b) on five consecutive occasions. The depletion efficiency of the MC-21 treatment was evaluated by flow cytometric analysis of peripheral blood samples^17^.

### Tissue preparation and immunofluorescence staining

To evaluate whether local administration of WORT alters expression of LC3B within the retina, eyes from treated mice were enucleated at various time points and cryosections prepared. Following fixation with 2% PFA and permeabilization with 0.1% Triton X-100, sections were blocked with 10% normal goat serum before incubation with a polyclonal rabbit anti-LC3B antibody (1:1000) and mouse anti-RPE65 mAb (1:200) overnight at 4 °C. After wash, sections were further incubated with goat anti-rabbit conjugated with Alexa Fluor 488 and goat anti-mouse conjugated with Alexa Fluor 555 (both 1:400). DAPI counterstain was used to show nuclei in sections.

Retinal cell apoptosis was determined by TUNEL staining using an *In Situ* Cell Death Detection Kit (Roche Diagnostics, Burgess Hill, UK) according to the manufacturer’s instructions.

To prepare RPE/Choroid whole-mounts, enucleated eyes were initially fixed in 2% PFA overnight. Eyes were dissected, and RPE/choroidal tissues were blocked and permeabilized in 5% BSA with 0.3% Triton X-100 in PBS for 2 hours, followed by incubation with a polyclonal rabbit anti-Iba1 (1:500) and goat anti-cleaved caspase-1 p20 (1:50) in 1% BSA with 0.15% Triton X-100 at 4 °C overnight. After thorough wash, samples were further incubated with secondary antibodies including DyLight^®^ 488-donkey anti-rabbit IgG and Alexa Fluor 405-donkey anti-goat IgG (both 1:200). Tissues were washed and flat-mounted in Vectashield antifade medium and examined by confocal microscopy.

For evaluation of CNV formation, neovascular membrane was stained with biotin-conjugated isolectin B4 (IB4, 1:100, Sigma Aldrich), followed by incubation with Rhodamine Red-X-labeled streptavidin (1:400, Jackson ImmunoResearch Laboratories, Suffolk, UK). The CNV volume was measured using a series of Z-stack images (from the surface to the deepest focal plane) using the Volocity® Image Analysis Software 6.0.

### Statistics

Results are presented as means ± SEM. Data were analyzed using Mann-Whitney test. Differences between groups were considered significant at P < 0.05.

## Additional Information

**How to cite this article**: Liu, J. *et al.* Impairing autophagy in retinal pigment epithelium leads to inflammasome activation and enhanced macrophage-mediated angiogenesis. *Sci. Rep.* **6**, 20639; doi: 10.1038/srep20639 (2016).

## Supplementary Material

Supplementary Information
