# Supplementary material for: Impairing autophagy in retinal pigment epithelium leads to inflammasome activation and enhanced macrophage-mediated angiogenesis
Source: Sci Rep. 2016 Feb 5;6:20639. doi: 10.1038/srep20639 (PMC4742917; doi:10.1038/srep20639)
Supplement: Supplementary Information [file srep20639-s1.pdf]

Supplementary Figures for Manuscript

Impairing autophagy in retinal pigment epithelium leads to inflammasome activation and enhanced macrophage-mediated angiogenesis

Jian Liu, David A. Copland, Sofia Theodoropoulou, Hsi An Amy Chiu, Miriam Durazo Barba, Ka Wang Mak, Matthias Mack, Lindsay B. Nicholson, Andrew D. Dick

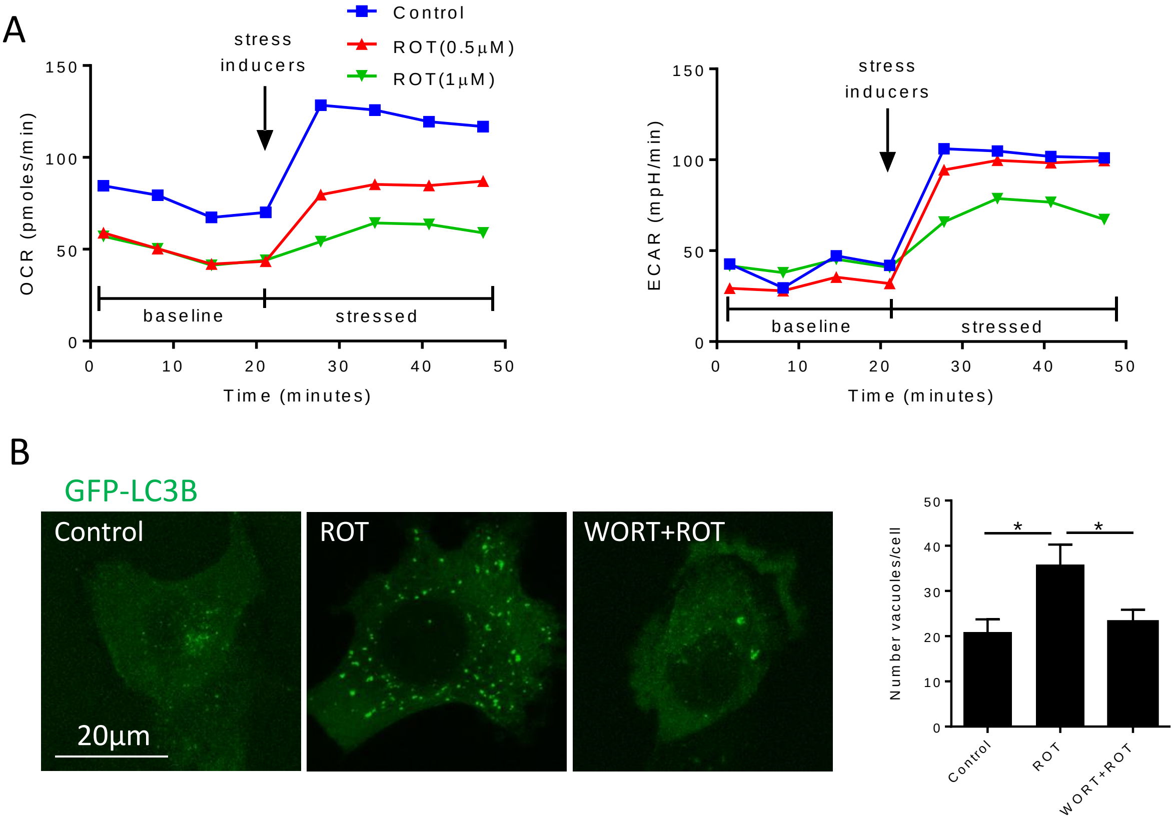

Supplementary Figure 1. Inhibition of mitochondrial damage-induced autophagy in B6-RPE07 cell culture. (A) ROT-caused changes in metabolic functionality indicated by oxygen consumption rate (OCR) and extracellular acidification rate (ECAR) in RPE cells were measured using an XFp Extracellular Flux Analyzer. (B) Representative confocal images and quantitative data show the activation of autophagy by ROT (0.5 µM) and its regulation by pretreatment with WORT (2 µM), using GFP-LC3B (a fluorescent autophagy marker) transduced RPE cells. n=30. $^{*}P<0.05$.

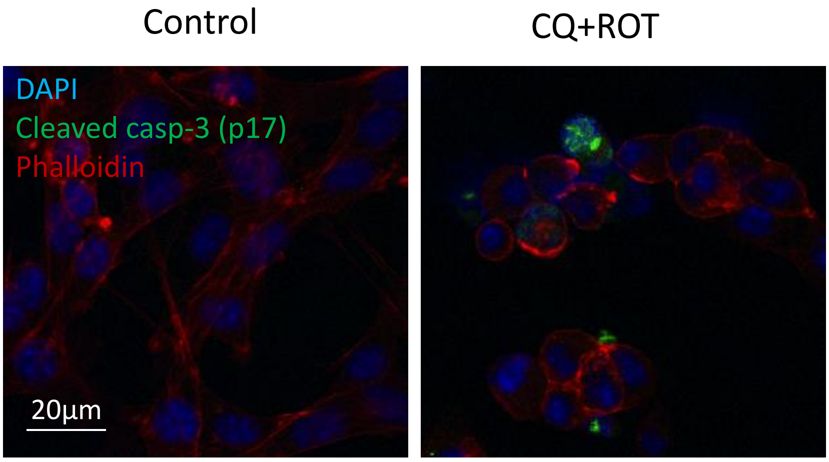

Supplementary Figure 2. Inhibition of ROT-induced autophagy in RPE cells leads to activation of caspase-3. RPE cells were pretreated with CQ (an downstream autophagy inhibitor) for 90 minutes, followed by addition of ROT. After 24 hours incubation, cells were fixed and permeabilized for immuno-staining with anti-cleaved caspase-3 (p17) antibody. DAPI and phalloidin were used to stain nuclei and cytoskeleton F-actin, respectively.

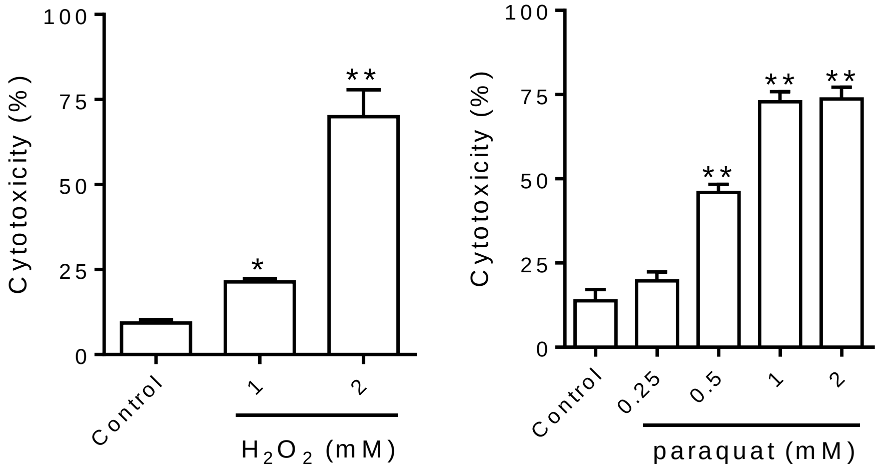

Supplementary Figure 3. Cytotoxicity of pro-oxidants in RPE cell cultures. B6-RPE07 cells were incubated with hydrogen peroxide (H_2O_2) or PQ with different concentrations for 48 hours. Cytotoxicity was assessed by LDH release in the cell culture supernatants. n≥3. $^{*}P<0.05$; $^{**}P<0.01$ vs. control.

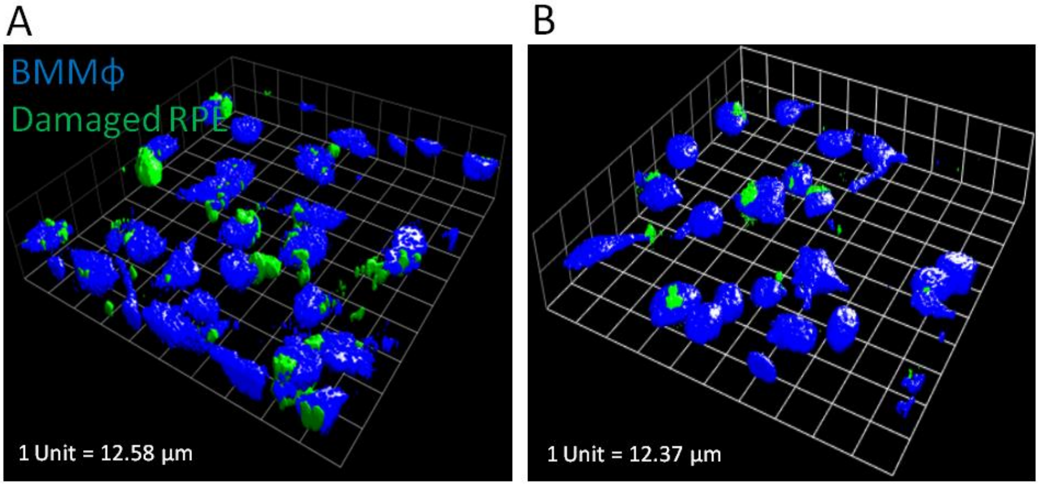

Supplementary Figure 4. 3D reconstruction of confocal images show engulfment of dysfunctional RPE cells by BMMΦs. Damaged RPE cells (CFDA-labeled) caused by WORT+ROT treatment were added to BMMΦs (Violet Tracer-labeled). BMMΦs can phagocytose damaged RPE cells/debris within 60 minutes. After 1 hour (A) or 2 hours (B) incubation, the cells were washed three times to remove un-engulfed RPE cells/debris, and samples were fixed and observed by confocal microscopy. Note that there were fewer RPE cell fragments after 2 hours than 1 hour incubation, due to digestion and degradation over time.

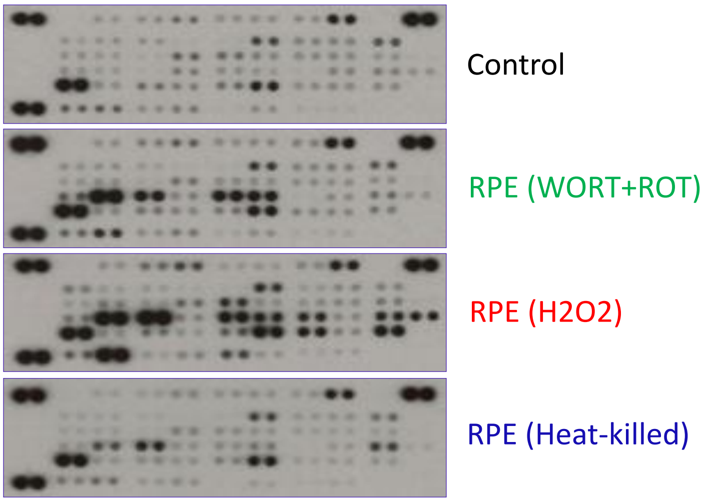

Supplementary Figure 5. Representative Mouse Angiogenesis Proteome Array membranes show the expression profile of angiogenesis-associated proteins secreted from BMMΦs treated with differently damaged RPE cells. Dead/dying RPE cell preparations include dysfunctional (WORT+ROT treated), oxidative stressed (H_2O_2 treated) and heat-killed cells (95°C heating for 15 minutes).

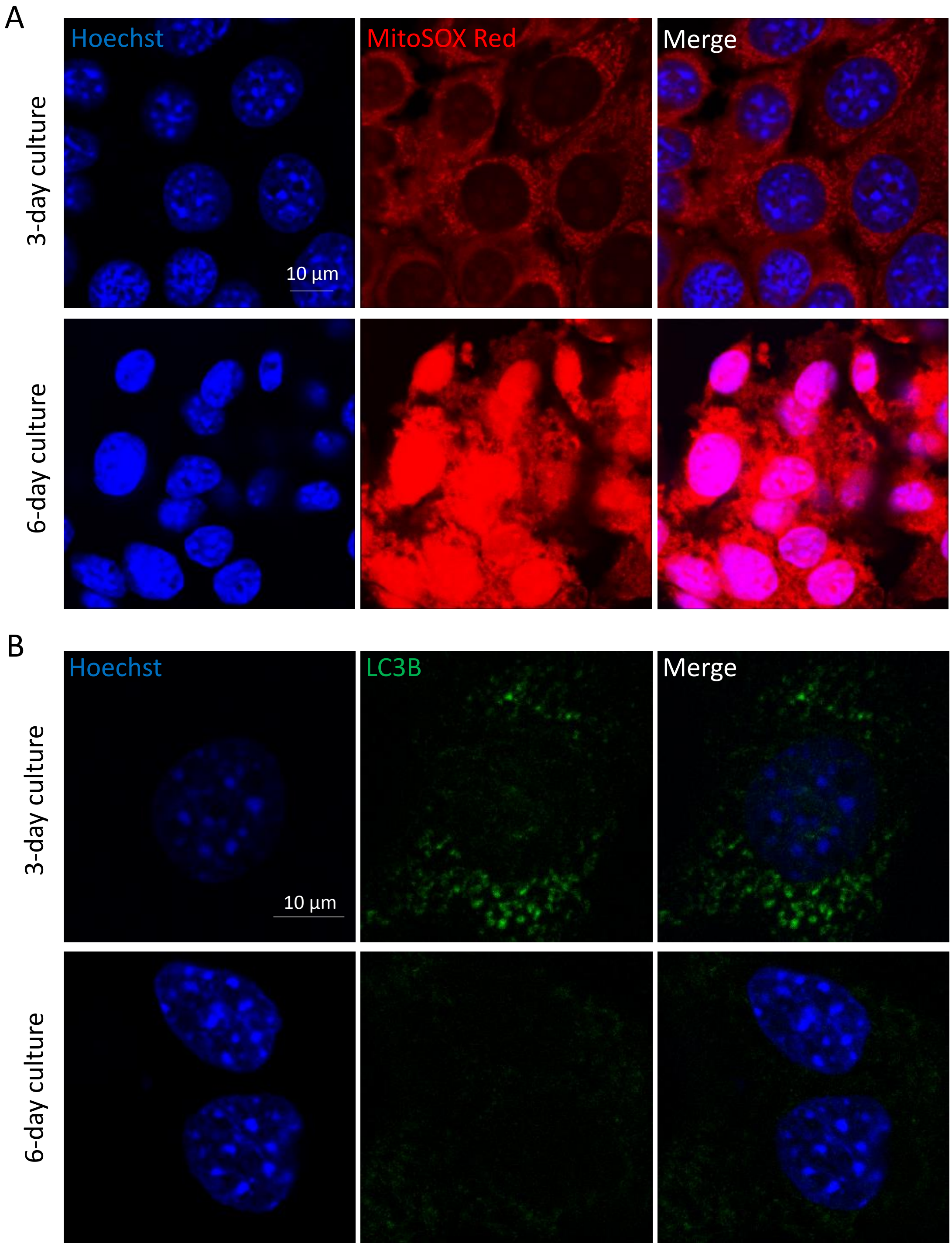
A
Hoechst
MitoSOX Red
Merge
3-day culture
6-day culture
10 µm
B
Hoechst
LC3B
Merge
3-day culture
6-day culture
10 µm

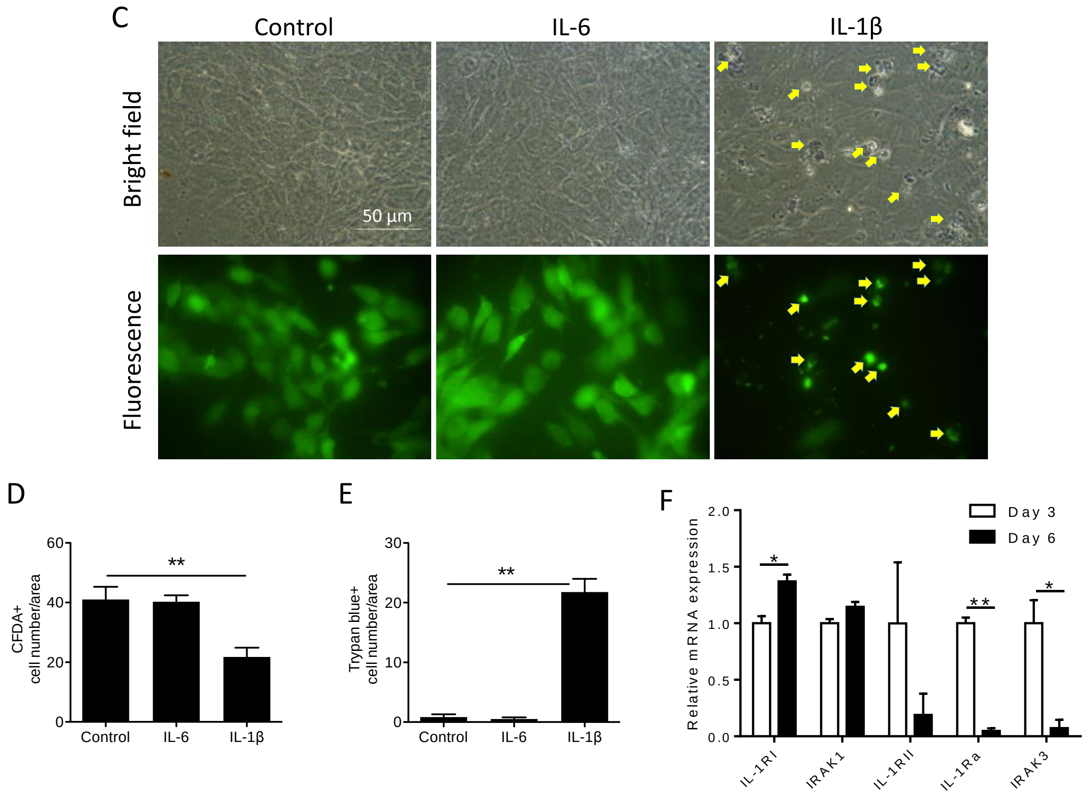

Supplementary Figure 6. Six-day cultured B6-RPE07 cells have damaged mitochondria and impaired autophagy, and are susceptible to IL-1β mediated cytotoxicity, with comparison to 3-day cultured cells. (A) 3-day freshly confluent and 6-day over-confluent cells were labeled with mitoSOX Red indicator to stain mitochondrial superoxide in live cells for observation using confocal microscopy. Hoechst 33342 was used for nuclear stain. 6-day cultures demonstrated strong nuclear staining of mitoSOX Red, indicating a loss of either mitochondrial structure or membrane potential generating highly fluorescent oxidized products that bind to nucleic acids. (B) 3-day and 6-day cell cultures were treated with 0.5 μM of ROT for 24 hours. The cells were immuno-stained for analysis of LC3B expression. (C) 6-day cultured RPE cells (CFDA labeled) and 3-day cells were mixed at ratio of 1:1 to form confluent monolayers, which were then treated with 2 ng/ml of IL-6 or IL-1β. After 72 hours, the co-culture was stained with Trypan Blue (TB) and then fixed by 2% PFA for observation. RPE monolayers contain non-fluorescent 3-day cultured cells and fluorescent 6-day cells. Live cells in the monolayers were identified by TB exclusion. Yellow arrows point to fluorescent cells with TB inclusion. Quantitative analysis shows mean number of CFDA^{+} (D) and TB^{+} cells (E). The results show that the 6-day cells can survive in the mixed culture with IL-6 treatment, while those were removed by IL-1β treatment. (F) RNA from 3-day and 6 day cultured RPE cells were analyzed for expression of genes involved in IL-1 signaling pathways. $n \geq 3$. $^{*}P<0.05$, $^{**}P<0.01$.

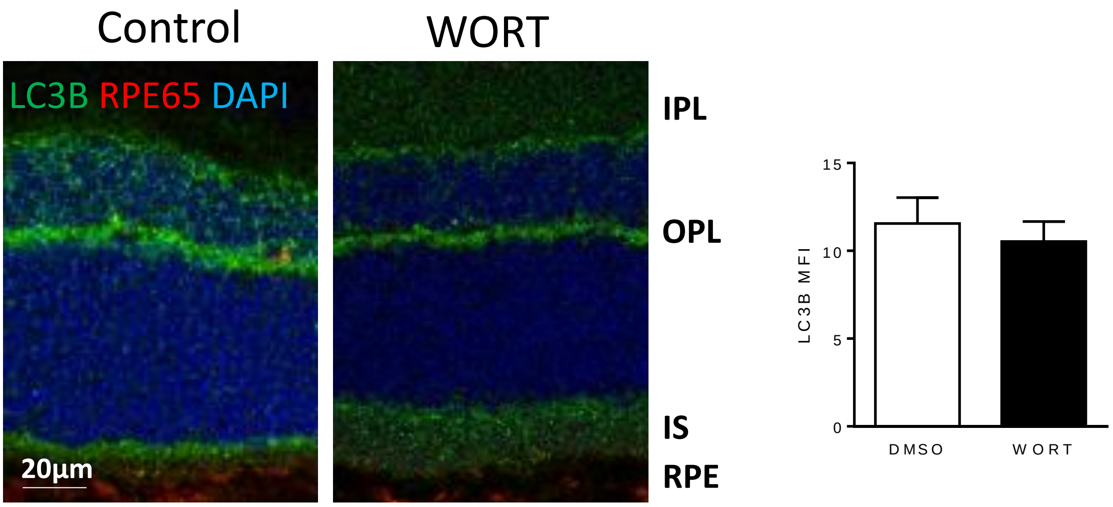

Supplementary Figure 7. Representative confocal images of sections show expression of LC3B in the retina seven days post injection with WORT or control. Quantitative LC3B-MFI analysis shows relative LC3B immuno-reactivity in retina after 7 days (n=4). RPE65 antibody and DAPI stain RPE and nuclei, respectively.

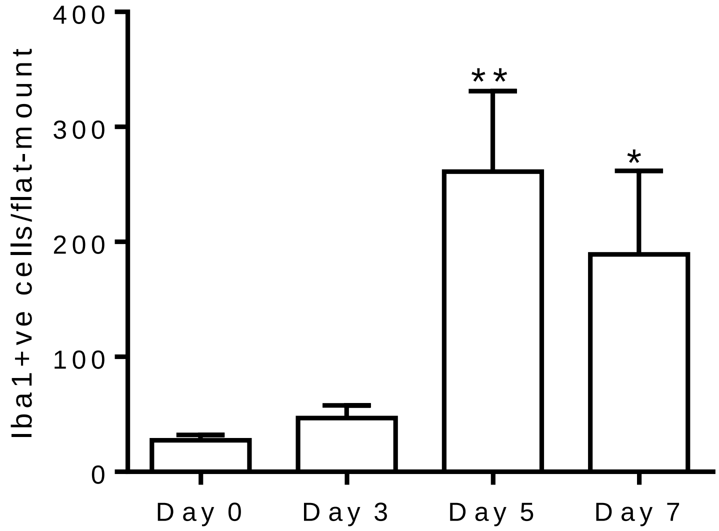

Supplementary Figure 8. Time-dependent accumulation of $Iba1^{+}$ macrophages to the sites of RPE degeneration at the sub-retinal space post WORT injection. At different time, eyes were collected and RPE/choroid flat-mounts prepared for immunofluorescence staining (see main Fig. 4C and D). $Iba1^{+}$ cells on the flat-mounts were observed and counted under the fluorescence microscopy. n=4 to 12. *P<0.05, **P<0.01 vs. Day 0.

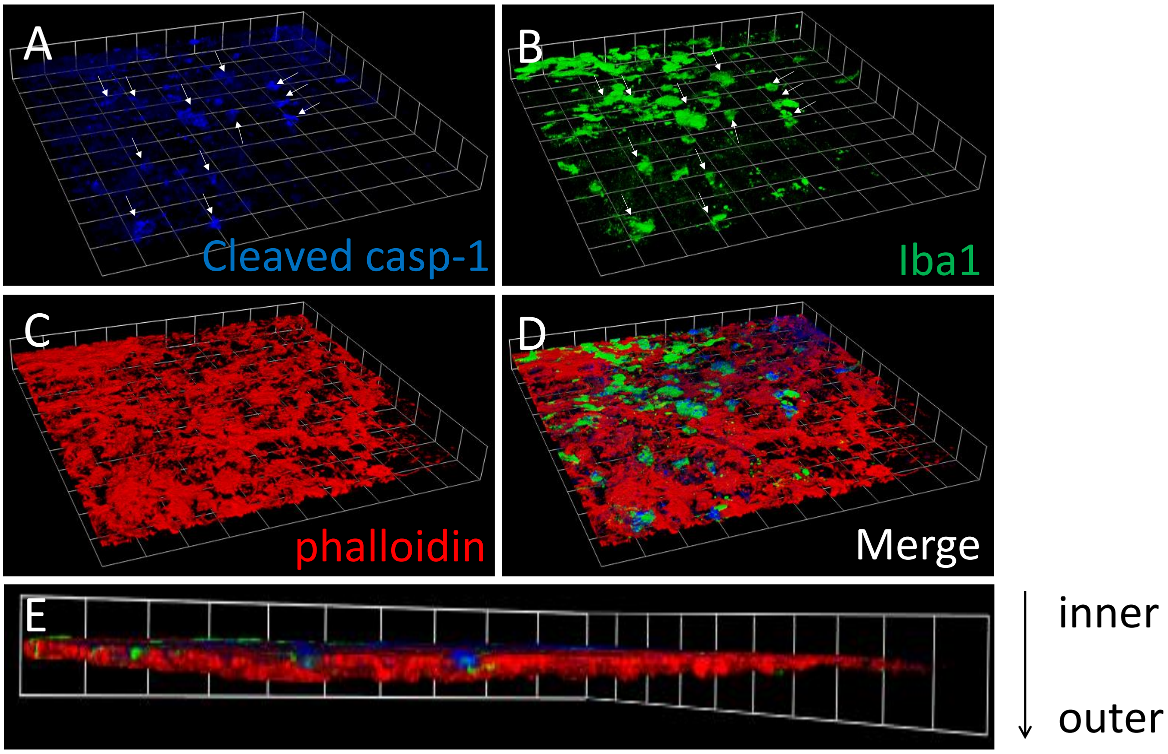

Supplementary Figure 9. 3D reconstruction of confocal images show expression of active caspase-1 (A), Iba1(B) and F-actin (C) on RPE/choroidal flat-mounts on day 5 post WORT injection. Merged color image is shown in D and E (side view). White arrows (A and B) demonstrate co-localisation of active caspase-1 with Iba1^{+} macrophages, which are on top of damaged RPE (E). 1 Unit=38.98 µm.

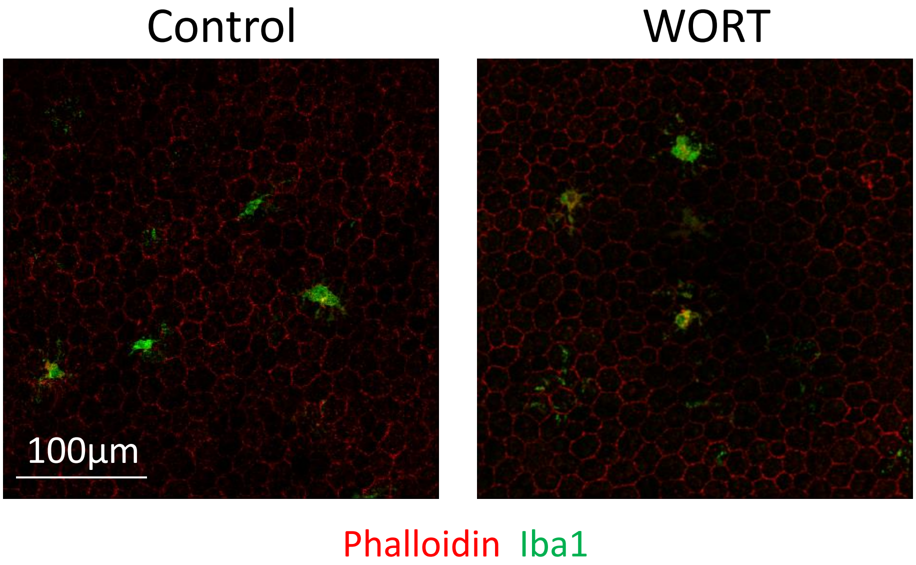

Supplementary Figure 10. Representative images of RPE/choroidal whole-mounts show the RPE monolayer (phalloidin stain) and macrophages (Iba1 stain) on day 12 post WORT injection.
